# Is utility-based quality of life associated with overweight in children? Evidence from the UK WAVES randomised controlled study

**DOI:** 10.1186/s12887-015-0526-1

**Published:** 2015-12-16

**Authors:** Emma J. Frew, Miranda Pallan, Emma Lancashire, Karla Hemming, Peymane Adab, on behalf of the WAVES Study co-investigators

**Affiliations:** Health Economics Unit, University of Birmingham, Birmingham, B15 2TT UK; Department of Public Health, Epidemiology and Biostatistics, School of Health and Populations Sciences, University of Birmingham, Birmingham, B15 2TT UK

**Keywords:** Health-related quality of life, Utility, CHU9D, BMI, Children, UK

## Abstract

**Background:**

Quality-Adjusted Life Years (QALYs) are often used to make judgements about the relative cost-effectiveness of competing interventions and require an understanding of the relationship between health and health-related quality of life (HRQOL) when measured in utility terms. There is a dearth of information in the literature concerning how childhood overweight is associated with quality of life when this is measured using utilities. This study explores how weight is associated with utility-based HRQOL in 5–6 year olds and examines the psychometric properties of a newly developed pediatric utility measure – the CHU9D instrument.

**Methods:**

Weight and HRQOL were examined using data collected from 1334 children recruited within a UK randomised controlled trial (WAVES) (ISRCTN97000586). Utility-based HRQOL was measured using the CHU9D, and general HRQOL measured using the PedsQL instrument. The association between weight and HRQOL was examined through a series of descriptive and multivariate analysis. The construct validity of the CHU9D was further assessed in relation to weight status, in direct comparison to the PedsQL instrument.

**Results:**

The HRQOL of children who were either overweight or obese was not statistically different from children who were healthy or underweight. This result was the same for when HRQOL was measured in utility terms using the CHU9D instrument, and in general terms using the PedsQL instrument. Furthermore, the results support the construct validity of the newly developed CHU9D as the PedsQL total HRQOL scores corresponded well with the individual CHU9D dimensions.

**Conclusion:**

At age 5–6 years, the inverse association between overweight and HRQOL is not being captured by either the utility-based CHU9D instrument nor the PedsQL instrument. This result has implications for how the cost-effectiveness of childhood obesity interventions is measured in children aged 5–6 years.

**Trial registration:**

ISRCTN Registry: ISRCTN97000586 19^th^ May 2010.

## Background

Childhood obesity is a growing problem worldwide [1–3]. The direct annual costs of obesity and associated health consequences across the EU is about 7 % of national health budgets [4] and within the UK National Health Service (NHS), is approximately £4.2 billion, with an estimated cost of £16 billion to the wider economy [5].

A range of interventions have been developed to prevent and manage childhood obesity [6]. However, there is an absence of evidence on the cost-effectiveness of such interventions. Whilst there is much evidence to suggest that weight status has an effect on adult health-related quality of life (HRQOL) [7–11], and many studies have reported similar associations in adolescents [12–14], these studies report HRQOL in general terms rather than in the more specific utility terms required for an economic analysis. In the UK, for decision making bodies such as the National Institute for Health and Care Excellence (NICE) it is recommended that HRQOL is measured in utility terms to facilitate the construction of Quality-Adjusted Life Years (QALYs). QALYs are then used as the unit of assessment for comparing the cost-effectiveness of alternative interventions [15] and are now used to inform resource allocation decisions worldwide [16]. Conventional practice within economic evaluations is to measure HRQOL on a cardinal 0–1 utility scale with death (0) and full health (1) denoting either end of the scale [17]. Very few studies have looked at the impact of childhood overweight/obesity on HRQOL when it is measured in utility terms [18] yet this information is vital for the construction of QALYs. This study directly addresses this evidence gap.

Assessment of health status in children differs from adults and requires a different conceptual approach due to rapid rates of development, dependency on parents/caregivers and differences in disease epidemiology [19]. Utility-based HRQOL in children therefore needs to be measured using an instrument specifically designed for children. The CHU9D is a recently developed generic HRQOL measure designed to produce utility information. Originally tested for 7–11 year olds [20, 21], it has more recently demonstrated good construct validity in adolescents aged 11–17 years [22]. Although there is emerging evidence regarding the psychometric properties of the CHU9D instrument [22, 23], more evidence is required with respect to its validity for use in different age groups and country settings. Different terms are used in the literature to describe validity, and in this context, discriminant validity refers to the degree with which the instrument discriminates between groups with known differences, and convergent validity refers to the degree to which two theoretically related measures of construct are actually related. Both are subtypes of construct validity [24].

This paper explored the relationship between weight status and utility-based HRQOL (measured on a 0–1 scale reflecting full health and death) in children aged 5–6 years. Also it examined the construct validity of the CHU9D instrument by reporting specifically on the discriminant and convergent validity. To facilitate this assessment, the CHU9D was directly compared to the PedsQL instrument [25], a widely used, validated generic HRQOL measure in children.

## Methods

The WAVES study is a UK-based cluster-randomised controlled trial assessing clinical and cost-effectiveness of an obesity prevention intervention targeting children, funded by the UK National Institute for Health Research (ISRCTN97000586; Date of registration: 19/5/2010) from 2010 to 2015. Fifty-four schools (recruited from a random sample of 200) participated in the study. The study had full ethics approval and was conducted in accordance with the World Medical Association’s Declaration of Helsinki (National Research Ethics Service Committee, West Midlands, The Black Country No. 10/H1202/69). The random sample was weighted to achieve sufficient representation (to enable sub group analysis) from the two most prevalent ethnic minority groups in the West Midlands, UK: South Asian (Bangladeshi, Indian and Pakistani) and Black (African and Caribbean). All children in school year 1 (aged 5–6) from participating schools were invited to take part. Written parental consent was obtained for each study participant through a signed consent form and verbal assent from the children at the point of measurement. Parental consent was obtained for 1470 children (60 % of those eligible), and 1401 children (95 % of those consented/57 % of those eligible) were available for baseline measurements. For practical reasons the schools were split into two groups, half the schools had baseline measurements taken in 2011 and the other half in 2012. Data on participants’ date of birth, sex and postcode were obtained from school records. Ethnicity data were collected through a parent completed questionnaire, or school records when this was not available. Small area deprivation was used as a proxy for socioeconomic status. Deprivation was assessed using the index of multiple deprivation (IMD) [26]. The IMD score for the residential area of each child was identified based on their postcode using an online facility [27]. These scores were then allocated to the appropriate IMD quintile; those in the first quintile, living in an area classified by the IMD as one of the 20 % most deprived in England and those in the 5^th^ in an area classified as one of the 20 % least deprived.

### Measurement of weight status

For all participants, height and weight measures were taken at school by trained researchers using standardised instruments and procedures. Height was measured to the nearest 0.1 cm using a Leicester height measure. Weight was measured in light clothing without shoes to the nearest 0.1 kg using a Tanita SC-331 S body composition analyser. BMI was calculated by dividing weight (in kilograms) by height (in metres) squared (kg/m2) and used to categorise the children into underweight, healthy weight, overweight and obese groups. The 2^nd^, 85^th^ and 95^th^ centiles of the UK 1990 Growth reference charts for BMI [28] were used to define the four weight categories, in line with standard UK definitions [29].

### HRQOL measures

As the focus of this study was to explore the association between weight status and HRQOL when measured in utility terms, two instruments were selected for the measurement of HRQOL. Both are generic instruments and thus are designed to measure a wider notion of HRQOL and are not specific to any one disease or condition. The CHU9D is a preference-based utility instrument designed exclusively for use in children and previous research has shown this instrument is the most appropriate choice in this age group [30]. As a utility-based instrument, it is designed to produce a HRQOL score that is preference-based and set between the values of 0 (death) and 1 (full health), however like many preference-based utility instruments, it does produce scores that are deemed to be ‘worse than death’ and therefore have values of less than 0. The PedsQL was chosen as a ‘gold standard’ comparator as this is a widely used HRQOL instrument validated for use in this age group and was the instrument of choice for the WAVES trial from which the data was generated. Although this instrument is non-utility based would be expected to generate HRQOL values which move in the same direction as the CHU9D utility values.

#### CHU9D

The CHU9D instrument contains 9 dimensions: school work/homework; tired; sleep; worried; sad; annoyed; daily routine; ability to join in activities; and pain, and every dimension contains 5 levels indicating the severity of the dimension. Each of the possible 1,953,125 unique health states are assigned a health utility value ranging from 0.33 to 1 based on an algorithm that reflects the preference weight attached to each dimension [31] .

#### PedsQL

The PedsQL is a 23-item instrument including four domains: physical (8 items), emotional (5 items), social (5 items), and school (5 items) functioning [25, 32]. For this study we used the child self-report PedsQL version designed for use in 5–7 year olds. Emerging from the instrument is a score (transformed on to a 0–100 scale) for each type of functioning, with higher scores indicating better quality of life. Each item has three response options: not at all; sometimes; a lot; which in the scoring process are assigned values of 100; 50; 0, respectively. Provided data are available for at least half of the relevant items, the mean score for each of the four domains is then calculated by summing the values for the relevant items and dividing by the number of items answered. This is repeated including all items for the total score. The PedsQL instrument has good reliability and validity in both sick and healthy populations [32–35].

Both the CHU9D and the PedsQL were administered at the same time point by researchers on a one-to-one basis. The items and possible responses were read out and to help the children understand how to answer, for the PedsQL, a visual prompt (of a face ranging from smiley to sad associated with each response option) was provided as recommended by the developers of the instrument for administration to young children.

#### Statistical analysis

In the absence of a gold standard for the measurement of utility-based HRQOL in young children, and with no prior knowledge of how weight status affects utility-based HRQOL in children, to measure the construct validity of the CHU9D, we looked at the relationship between CHU9D and PedsQL in relation to weight status. This method allowed us to explore two subtypes of construct validity: discriminant and convergent validity. We explored discriminant validity by determining if the CHU9D instrument was able to discriminate between children within different weight groups, and the convergent validity by assessing how the CHU9D correlated with the PedsQL measure.

To explore the relationship between HRQOL and sample characteristics we report mean (and SD) CHU9D and PedsQL scores by weight status category, gender, ethnic group and deprivation quintile. Differences in HRQOL scores between groups were assessed using either the Kruskal-Wallis test, or the non-parametric test for trend. To examine the construct validity of the CHU9D, we split the sample according to the median PedsQL total score and examined separately the mean CHU9D utility value for children who scored on or above this median score, and those who scored below it. This difference was then compared using the one-way ANOVA test. Next, we looked at the distribution of response to each of the CHU9D dimensions by weight status category to assess if there were any significant differences in response. We hypothesised that children in the overweight and obese category would report more problems in each dimension compared to children in the healthy and underweight category. We assessed the significance of differences in response using the chi-squared test. To determine how well the PedsQL scores correspond with the CHU9D dimensions we estimated the mean PedsQL total score for each level of CHU9D response with the expectation that with increasing severity on each CHU9D dimension, the mean PedsQL total score would be lower. A scatter plot (along with fitted regression line and 95 % CIs) for the CHU9D utility values and the total PedsQL scores was used to visualise the correlation between the instruments, and the correlation coefficient was calculated using the Spearman’s rho statistic. To explore the correlation further we looked at the relationship between theoretically similar dimensions within both instruments. Our prior expectation was that the following dimensions would be correlated:

**Table:** 

**PedsQL Instrument**	**CHU9D instrument**
Physical functioning	Tired/Able to join in activities/Daily routine/Pain/Sleep
Emotional functioning	Sad/Annoyed/Worried
Social functioning	Able to join in activities
School functioning	School work/home work

Finally, to compare the CHU9D utility values between the weight groups we used a linear mixed regression model (with random effect for school), adjusted for potential confounders (age, gender, ethnicity and deprivation quintile). All analyses were undertaken in 2014, using Stata version 13.

## Results

Full data (including PedsQL total score, CHU9D utility value, and weight status group) were available for 1344 children and are presented in Table 1. The proportion of children in the study sample who were either obese or overweight (21.7 %) is similar to the most comparable national data available [36] in which 22.6 % of children measured in their Reception Year during the 2011/12 school year were classified as overweight or obese.

**Table 1:** Sample Characteristics

Characteristics	
Gender: n (%) (*n* = 1344)	
Male	695 (51.7)
Female	649 (48.3)
Age: mean (SD) (*n* = 1344)	6.3 (0.31)
Ethnic origin: n (%) (*n* = 1328)	
White British	603 (45.4)
South Asian	403 (30.3)
African Caribbean	107 (8.1)
Other	215 (16.2)
Deprivation quintile: n (%) (*n* = 1324)	
1 Most deprived	738 (55.8)
2	239 (18.1)
3	146 (11.0)
4	113 (8.5)
5 Least deprived	88 (6.6)
Weight: n (%) (*n* = 1344)	
Underweight	40 (3.0)
Healthy weight	1012 (75.3)
Overweight	116 (8.6)
Obese	176 (13.1)
CHU9D mean score (SD) (*n* = 1344)	0.825 (0.14)
PedsQL mean score (SD):	
PedsQL Physical functioning (*n* = 1344)	74.03 (17.56)
PedsQL Emotional functioning (*n* = 1344)	72.32 (22.74)
PedsQL Social functioning (*n* = 1344)	68.11 (22.23)
PedsQL School functioning (*n* = 1344)	67.15 (21.89)
PedsQL Psychosocial functioning (*n* = 1344)	68.93 (18.13)
PedsQL Total scale score (*n* = 1344)	70.44 (16.04)

### Discriminant validity

Using the known-groups method, the CHU9D (but not the PedsQL) differentiated HRQOL in children of different ethnic origin (*p* =0.028) with White British children having the highest mean utility score (Table 2). There was a statistically significant trend of decreasing HRQOL by increasing level of deprivation which was identified by both instruments (*P* < 0.05). When children were categorised into two groups according to their weight status, neither instrument differentiated between the two groups.

**Table 2:** Mean CHU9D and PedsQL scores grouped by respondent characteristics

	Number	Mean CHU9D Utility (SD)	PEDSQL total score (SD)
Gender			
Male	695	0.826 (0.14)	71.10 (16.81)
Female	649	0.824 (0.13)	69.72 (15.17)
*p* ^a^		0.38	0.05
Ethnic Origin:			
White British	603	0.836 (0.13)	71.41 (16.07)
Asian	403	0.809 (0.15)	69.19 (15.66)
African Carribean	107	0.818 (0.15)	69.18 (18.35)
Other	215	0.822 (0.12)	70.63 (15.27)
*p* ^a^		0.02	0.09
Weight status groups:			
Underweight	40	0.851 (0.13)	72.52 (17.51)
Normal weight	1012	0.825 (0.14)	70.81 (15.57)
Overweight	116	0.811 (0.14)	67.97 (16.12)
Obese	176	0.827 (0.13)	69.44 (18.13)
*p* ^b^		0.33	0.28
Weight status groups:			
Underweight/Healthy weight	1052	0.83 (0.14)	70.87 (15.64)
Overweight/obese	292	0.82 (0.13)	68.86 (17.35)
*p* ^b^		0.30	0.18
Deprivation quintiles:			
1 Most deprived	738	0.81 (0.14)	69.17 (16.28)
2	239	0.81 (0.14)	71.14 (16.10)
3	146	0.84 (0.13)	73.04 (15.19)
4	113	0.82 (0.13)	71.48 (15.98)
5 Least deprived	88	0.86 (0.11)	72.97 (14.45)
p^b^		<0.001	0.002

^a^Kruskal-Wallis test; ^b^non-parametric test for trend

To explore the discriminant validity of the CHU9D instrument, the mean and standard deviations for the CHU9D utility values were estimated for children who had a score either above, or below, the median PedsQL total score (71.73) for the sample. The mean utility scores were 0.87 (SD 0.109) and 0.76 (SD 0.143) respectively (*p* < 0.001).

Table 3 shows the distribution of the CHU9D dimensions by weight status category. Overall, the majority of children had no or few problems for all dimensions, irrespective of weight status. There were no underlying differences in the distribution of response to any of the CHU9D dimensions between children in the different weight categories.

**Table 3:** Distribution of response to CHU9D dimensions by weight status category

CHU9D Dimensions	Level	Healthy and underweight (*n* = 1052)	Overweight and obese (*n* = 292)	Chi-squared test
		n (%)	n (%)	*p*
Worried	No	649 (61.7)	187 (64.0)	0.68
	A little bit	171 (16.3)	46 (15.8)	
	A bit	70 (6.6)	23 (7.9)	
	Quite	67 (6.4)	14 (4.8)	
	Very	95 (9.0)	22 (7.5)	
Sad	No	669 (63.6)	181 (62.0)	0.84
	A little bit	168 (16.0)	48 (16.4)	
	A bit	61 (5.8)	14 (4.8)	
	Quite	86 (8.1)	29 (10.0)	
	Very	68 (6.5)	20 (6.8)	
Pain	No	665 (63.2)	187 (64.0)	0.66
	A little bit	191 (18.2)	51 (17.5)	
	A bit	56 (5.3)	10 (3.4)	
	Quite	47 (4.5)	16 (5.5)	
	Very	93 (8.8)	28 (9.6)	
Tired	No	492 (46.8)	141 (48.3)	0.37
	A little bit	183 (17.4)	61 (20.9)	
	A bit	93 (8.8)	18 (6.1)	
	Quite	69 (6.6)	16 (5.5)	
	Very	215 (20.4)	56 (19.2)	
Annoyed	No	718 (68.2)	196 (67.1)	0.09
	A little bit	117 (11.1)	28 (9.6)	
	A bit	55 (5.2)	19 (6.5)	
	Quite	40 (3.8)	21 (7.2)	
	Very	122 (11.6)	28 (9.6)	
School/home work	No problems	622 (59.1)	186 (63.7)	0.37
	A few problems	185 (17.6)	45 (15.4)	
	Some problems	94 (9.0)	24 (8.2)	
	Many problems	49 (4.6)	17 (5.8)	
	Can’t do	102 (9.7)	20 (6.9)	
Sleep	No problems	549 (52.2)	135 (46.2)	0.17
	A few problems	140 (13.3)	38 (13.0)	
	Some problems	73 (7.0)	18 (6.1)	
	Many problems	45 (4.2)	19 (6.5)	
	Can’t do	245 (23.3)	82 (28.0)	
Daily routine	No problems	741 (70.4)	198 (67.8)	0.48
	A few problems	113 (10.7)	41 (14.0)	
	Some problems	77 (7.3)	20 (6.9)	
	Many problems	40 (3.8)	14 (4.8)	
	Can’t do	81 (7.7)	19 (6.5)	
Activities	Any	723 (68.7)	189 (64.7)	0.28
	Most	136 (12.9)	48 (16.4)	
	Some	79 (7.5)	21 (7.2)	
	A few	61 (5.8)	23 (7.9)	
	No	53 (5.1)	11 (3.8)	

Table 4 shows how the mean PedsQL scores corresponded with the options for each of the CHU9D dimensions. The mean PedsQL total scores decrease linearly with increasing severity on each of the CHU9D dimensions.

**Table 4:** Mean PedsQL score by each level of CHU9D dimension

CHU9D Dimensions	Level	n	Mean PedsQL score (SD)	*p* ^a^
Worried	No	836	73.2 (15.05)	
	A little bit	217	68.0 (16.14)	
	A bit	93	68.6 (15.70)	
	Quite	81	65.6 (14.89)	
	Very	117	59.4 (17.53)	<0.001
Sad	No	850	72.9 (15.10)	<0.001
	A little bit	216	68.0 (15.95)	
	A bit	75	66.6 (15.69)	
	Quite	115	65.8 (16.37)	
	Very	88	60.8 (18.66)	
Pain	No	852	72.7 (15.65)	<0.001
	A little bit	242	69.4 (14.81)	
	A bit	66	69.5 (12.63)	
	Quite	63	63.5 (16.33)	
	Very	121	60.0 (17.39)	
Tired	No	633	75.2 (15.17)	<0.001
	A little bit	244	69.0 (13.84)	
	A bit	111	67.6 (16.60)	
	Quite	85	67.5 (16.40)	
	Very	271	62.5 (15.74)	
Annoyed	No	914	73.1 (15.43)	
	A little bit	145	66.5 (15.10)	
	A bit	74	65.0 (15.71)	
	Quite	61	65.2 (16.41)	
	Very	150	62.3 (16.11)	<0.001
School/home work	No problems	808	74.2 (14.98)	<0.001
	A few problems	230	67.7 (14.23)	
	Some problems	118	63.8 (16.75)	
	Many problems	66	62.0 (16.33)	
	Can’t do	122	60.9 (16.67)	
Sleep	No problems	684	74.8 (15.78)	<0.001
	A few problems	178	69.9 (11.91)	
	Some problems	91	64.0 (13.81)	
	Many problems	64	66.2 (14.21)	
	Can’t do	327	63.9 (6.47)	
Daily routine	No problems	939	73.7 (14.92)	<0.001
	A few problems	154	65.9 (15.19)	
	Some problems	97	65.2 (15.63)	
	Many problems	54	59.3 (15.57)	
	Can’t do	100	56.8 (15.57)	
Activities	Any	912	72.5 (15.68)	<0.001
	Most	184	69.0 (16.28)	
	Some	100	67.0 (13.76)	
	A few	84	64.1 (15.62)	
	No	64	58.0 (15.68)	

^a^Non-parametric test for trend

### Convergent validity

**Fig. 1:**
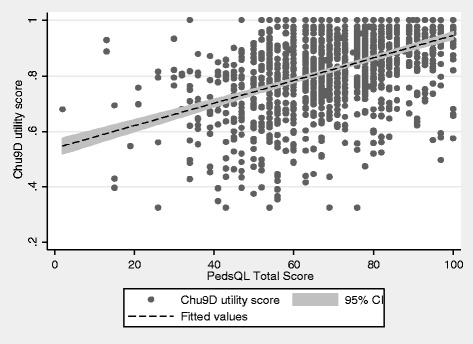
Relationship between CHU9D utility scores and PedsQL total scores

Figure 1 shows the relationship between the CHU9D utility values and the PedsQL total scores. Although there is a moderate association between the instruments with higher CHU9D utility values corresponding with higher PedsQL total scores, there are some anomalies. For example, one child reported a CHU9D utility of 0.32, yet had a PedsQL total score of 76.09, and another child reported a CHU9D utility score of 0.9, yet had a PedsQL total score of 13.04.

Overall, the correlation between the CHU9D utility values and PedsQL total scores showed a statistically significant moderate, positive correlation (rs = .4696, *p* = <0.001). The content and coverage of the two instruments were further assessed by examining the correlation between individual CHU9D dimensions and the theoretically similar PedsQL domains (Table 5).

**Table 5:** Correlation between CHU9D dimensions and PedsQL domain functioning scores

CHU9D dimension	Correlation with PedsQL score	Spearman’s ρ ^a^
Utility score	PedsQL total score	0.47
Worried	Emotional functioning	−0.18
Sad	Emotional functioning	−0.18
Pain	Physical functioning	−0.18
Tired	Physical functioning	−0.26
Annoyed	Emotional functioning	−0.22
School work/home work	School functioning	−0.21
Sleep	Physical functioning	−0.22
Daily routine	Physical functioning	−0.28
Able to join in activities	Social functioning	−0.13

^a^All were significant at 0.01 level

Using conventional cut-off values for Spearman’s ρ, we found that each CHU9D dimension was either weakly, or very weakly correlated with each of the pre-determined PedsQL domain functioning scores. As the CHU9D dimensions are coded with 1 as highest level and 5 as lowest level, the signs on the coefficients were consistently negative.

Table 6 shows the results of the linear mixed regression model (with random effect for school) which compared the CHU9D utility score between the two weight status groups, adjusted for potential confounders (age, gender, ethnicity and deprivation quintile). Children who are overweight or obese have a lower CHU9D utility value (i.e. poorer HRQOL) but this association is not statistically significant. Children from a non-White British background have lower mean CHU9D utility values and this association approaches significance (*p* = 0.07) for the South Asian population. Also, children from the least deprived areas have significantly higher CHU9D utility values relative to children from the most deprived areas.

**Table 6:** Results of linear mixed model to estimate variation in CHU9D between weight groups

Variables	Mean difference	95 % confidence intervals	*P*-value
Mean value	0.685	(0.529,0.841)	<0.001
Weight			
Underweight/Healthy weight			
Overweight/Obese	-0.005	(-0.023,0.012)	0.52
Age (years)	0.022	(-0.002,0.046)	0.07
Ethnic Group:			
White British	-		
South Asian	−0.019	(−0.040,0.002)	0.07
African-Caribbean	−0.006	(−0.037,0.239)	0.66
Other	−0.005	(−0.028,0.185)	0.66
Deprivation quintile:			
1 Most deprived			
2	0.001	(-0.021,0.024)	0.88
3	0.019	(-0.007,0.047)	0.15
4	-0.000	(-0.031,0.031)	0.99
Least deprived	0.040	(0.003,0.077)	0.03

## Discussion

Weight management interventions increasingly target preadolescent children and this has implications for the methods of outcome measurement within economic evaluation as few instruments exist that are designed to elicit utilities in this age group. This paper contributes evidence on the use of the newly developed utility-based CHU9D instrument, within an ethnically and socioeconomically diverse UK population of young children.

### Relationship between CHU9D and weight status

The results indicate that there is no statistically significant relationship between the CHU9D utility values and weight status in children aged 5–6 years. Adjusted for potential confounding factors, compared to the healthy/underweight group, children who were overweight/obese reported lower CHU9D utility values, but this effect was not statistically significant. A similar result was found using the PedsQL. When focusing on the CHU9D dimensions, there were no statistically significant differences in scores by child weight status group for any of the dimensions.

Four previous studies that have measured utility-based HRQOL in children [18, 37–39] have shown similar findings. In a US-based study, Belfort et al. (2011) used the Health Utilities Index-2 (HUI-2) instrument to measure utility-based HRQOL in children and adolescents aged 5–18 years, and found that utility scores were, on average, 0.04 lower in overweight/obese participants compared with healthy weight [37]. Boyle et al. (2010) used the EQ-5D-Y to investigate the effect of weight on the HRQOL in a UK-based population aged 11–15 years and found children who were overweight or obese had a significantly lower HRQOL than children of healthy weight [38]. A recently published paper explored the relationship between BMI and HRQOL using CHU9D in two cohorts of Australian children, aged 9–12 years and 14–16 years. They found mean CHU9D utility values to be lower in children who were overweight or obese (compared to ‘healthy’ weight children), but this effect was only significant in the younger age group [39]. Despite these reports of a negative relationship between HRQOL and being overweight in children, the evidence is mixed in terms of whether this effect reaches statistical significance. Within a UK-based pilot study that was linked to this study, the same direction of effect was found, but there was no statistical difference between utility values and weight status groups in children aged 5–6 years [18]. Three reasons were offered to help explain this result. The first related to the small pilot sample (*n* = 160), that may not have been large enough to assess subgroup differences. The sample size within this study population is substantially higher, and a similar result was found. The second reason suggested that the CHU9D is not sensitive enough to detect a difference in utility-based HRQOL between overweight and non-overweight children. In this study, the PedsQL total scores are available for comparison, and although the PedsQL shows a negative relationship between weight and HRQOL, again this does not reach statistical significance. Thirdly it was suggested that within this age group, the co-morbidities attached to obesity do not substantially affect HRQOL when measured on a 0–1 utility scale, and it is only once these children approach adolescence that the effects of being overweight have a negative impact on utility values. This might help explain the results within this study.

### Psychometric properties of CHU9D

This study has also contributed evidence on the construct validity of the CHU9D and the results support the convergent and the discriminant validity of the instrument. The most significant, consistent finding within the study population was that HRQOL when measured using both the CHU9D and the PedsQL, was lower within children from the most deprived areas, compared to children from the least deprived areas. This demonstrates that both instruments are discriminating between these groups of children with known differences. Also with respect to discriminant validity, the results showed that the mean CHU9D values were significantly higher for all children with a PedsQL total score greater than or equal to the sample median total PedsQL score, compared to children with a PedsQL total score less than the sample median. Furthermore, PedsQL total scores corresponded well with the individual CHU9D dimensions, with a lower mean PedsQL total score with increasing severity on each CHU9D dimension. Regarding the convergent validity, overall, there was a moderate, statistically significant positive correlation between the PedsQL total scores and the CHU9D utility values. However, despite this correlation between the overall scores of both instruments, we found only a weak, or very weak correlation between the dimensions of each instrument that were pre-determined as being theoretically similar. One possible explanation is that although the PedsQL total scores and the CHU9D utility values tap into a similar underlying construct (HRQOL), the individual dimensions of each instrument, while appearing quite similar, might actually be describing something that is quite specific and different. So at the dimension level the correlations are weak but when combined, the overall instrument scores become moderately correlated.

### Strengths and weaknesses of the study

The data within this study was collected from the WAVES trial which was designed to include a diverse socioeconomic and multi-ethnic population. Parental consent for participation in the WAVES trial was obtained for 57 % of eligible pupils which could lead to sample selection bias. However when the proportion consented out of those eligible was considered by several socio-demographic characteristics, although there was some variation, the differences were generally modest (sex (boys = 65 %, girls = 67 %), ethnicity (white = 75 %, South Asian = 61 %, Black African Caribbean = 64 %; deprivation (most deprived quintile = 65 %, least deprived quintile = 72 %).

As it is rare to have utility information available for children as young as 5 years and for this to be reported for different weight groups, this study contributes this much needed evidence. There are some limitations to note however. First, this paper reports data from a trial and the available data therefore were restricted to what was collected as part of the trial. Ideally, it would have been interesting to assess the convergent validity of the CHU9D utility data with HRQOL data collected using an obesity-specific HRQOL instrument. This would have allowed us to determine if the weak association between weight and utility-based HRQOL in this age group was due to there being no underlying relationship there at all or a lack of sensitivity with detecting the negative effects of being overweight through use of a generic instrument. However, the PedsQL is widely viewed as a ‘gold standard’ generic measure of HRQOL, and has been validated and used in diverse populations. We suggest this as an area for future research. Second, all questions within the PedsQL and the CHU9D were read out to children by an interviewer and this might have had an influence on how children responded. This was a pragmatic decision as children in this age group have very different reading abilities making self-completion problematic but it could have influenced children’s responses to the questions. Third, because of the very small number of children who were measuring ‘underweight’ in our sample (3 %) a decision was made to pull the ‘underweight’ and ‘healthy’ weight children into one weight category. There is no a priori reason to assume that the HRQOL of underweight and healthy weight children are similar but we could not explore this in a statistically robust fashion and the focus of this paper was on the effects of being overweight on HRQOL, not underweight. To enable a comprehensive analysis of the effects of being underweight would have required a purposive sampling approach to ensure adequate numbers of children in this category.

## Conclusion

This paper contributes utility data from a large UK-based pediatric population alongside information on the psychometric properties of the instrument used to generate these data. Studies suggest that overweight is negatively associated with HRQOL in children but the extent of the association, how it varies across age groups, and how it translates to the 0–1 utility scale is as yet under-researched. This paper offers support for the convergent and discriminant validity of the CHU9D, as a measure of utility-based HRQOL in children aged 5–6 years. It offers evidence that overweight is negatively associated with HRQOL in children in this young age group but that this association is weak. Utility values are frequently used within health economic studies conducted globally to derive QALYs to inform resource allocation decisions. Future studies need to determine how weight status is associated with HRQOL in utility terms, in different age cohorts, and across different country settings, to help inform the methods of economic evaluations alongside clinical trials of childhood obesity prevention and management.

## Abbreviations

BMI: Body mass index
CHU9D: Child Health Utility 9D
HRQOL: health-related quality of life
HUI: Health Utilities Index
NICE: National Institute for Health and Care Excellence
PedsQL: Pediatric Quality of Life Inventory TM
QALYs: quality adjusted life years
WAVES: The West Midlands ActiVe lifestyle and healthy Eating in School children study
